# Intranasal and epicutaneous administration of Toll-like receptor 7 (TLR7) agonists provides protection against influenza A virus-induced morbidity in mice

**DOI:** 10.1038/s41598-019-38864-5

**Published:** 2019-02-20

**Authors:** Eunice E. To, Jonathan Erlich, Felicia Liong, Raymond Luong, Stella Liong, Steven Bozinovski, Huei Jiunn Seow, John J. O’Leary, Doug A. Brooks, Ross Vlahos, Stavros Selemidis

**Affiliations:** 1 Oxidant and Inflammation Biology Group, School of Health and Biomedical Sciences, RMIT University, Bundoora, Victoria, 3083 Australia; 2 Department of Pharmacology, Infection and Immunity Program, Biomedicine Discovery Institute, Monash University, Clayton, Victoria, 3800 Australia; 3 Airways Inflammation Research Group, School of Health and Biomedical Sciences, RMIT University, Bundoora, Victoria, 3083 Australia; 4 Respiratory Research Group, School of Health and Biomedical Sciences, RMIT University, Bundoora, Victoria, 3083 Australia; 5 Discipline of Histopathology, School of Medicine, Trinity Translational Medicine Institute (TTMI), Trinity College Dublin, Dublin, Ireland; 6 Sir Patrick Dun’s Laboratory, Central Pathology Laboratory, St James’s Hospital, Dublin 8, Ireland; 7 Emer Casey Research Laboratory, Molecular Pathology Laboratory, The Coombe Women and Infants University Hospital, Dublin 8, Ireland; 8 CERVIVA research consortium, Trinity College Dublin, Dublin, Ireland; 9 School of Pharmacy and Medical Sciences, Division of Health Sciences, University of South Australia, Adelaide, 5001 Australia

## Abstract

Toll-like receptor 7 (TLR7) is a pattern recognition receptor that recognizes viral RNA following endocytosis of the virus and initiates a powerful immune response characterized by Type I IFN production and pro-inflammatory cytokine production. Despite this immune response, the virus causes very significant pathology, which may be inflammation-dependent. In the present study, we examined the effect of intranasal delivery of the TLR7 agonist, imiquimod or its topical formulation Aldara, on the inflammation and pathogenesis caused by IAV infection. In mice, daily intranasal delivery of imiquimod prevented peak viral replication, bodyweight loss, airway and pulmonary inflammation, and lung neutrophils. Imiquimod treatment also resulted in a significant reduction in pro-inflammatory neutrophil chemotactic cytokines and prevented the increase in viral-induced lung dysfunction. Various antibody isotypes (IgG1, IgG2a, total IgG, IgE and IgM), which were increased in the BALF following influenza A virus infection, were further increased with imiquimod. While epicutaneous application of Aldara had a significant effect on body weight, it did not reduce neutrophil and eosinophil airway infiltration; indicating less effective drug delivery for this formulation. We concluded that intranasal imiquimod facilitates a more effective immune response, which can limit the pathology associated with influenza A virus infection.

## Introduction

Influenza A virus (IAV) infections are a substantial global burden, resulting in significant morbidity and mortality^1^. The current prophylactic treatment strategies include vaccines and antivirals, but both of these have limitations that reduce their impact on viral pathogenesis. For example, vaccines provide very little protection against new or emerging strains of viruses that enter the population. Antivirals can be effective in alleviating clinical symptoms of IAV infection, but usually have a narrow window of administration; they can cause adverse effects; and are subject to strain resistance^2–4^. There is therefore a defined need for alternative therapeutic approaches that can offer protection against influenza viruses, regardless of the strain or pathogenicity.

Toll-like receptors (TLR) are a class of pattern recognition receptors (PRRs) that detect pathogen-associated molecular patterns (PAMPs)^5^. TLRs are found on antigen presenting cells such as macrophages, dendritic cells and B-cells, and are critical in initiating an innate immune response, which include cytokine and chemokine release^6^. TLR ligands (TLR3, TLR4, TLR7 and TLR9) have been employed to enhance immunogenicity against influenza virus infections^7,8^. However, these studies have not discerned if intranasal administration of the ligands can alleviate the clinical symptoms. Since genomic ssRNA that is released from the influenza virion is detected by TLR7^9^, this present study focuses on examining the protective role of a TLR7 agonist against influenza infection. Activation of TLR7 and the adaptor protein MyD88 by IAV causes stimulation of Type I interferons (IFN), IL-6 and IL-1β, cytokines which are generally thought to be protective^10^. However, despite this vigorous immune reaction there is progressive viral pathogenesis, which raises questions about the effectiveness of this immune response. The imidazoquinoline compound imiquimod, which is a TLR7 agonist appears to have anti-viral properties and has been used to treat viral infection associated with genital warts. Imiquimod has also been shown to be an effective adjuvant in influenza vaccines; with mice that were given imiquimod in combination with vaccination 3 days prior to a lethal dose of mouse adapted A(H1N1) pdm09 virus having a survival rate of 60% compared to 30%, 5% and 0% for vaccine-alone, imiquimod-alone and PBS control, respectively^11^. In addition, randomized controlled trials showed that with administration of imiquimod and intradermal influenza vaccinations, there was a 98% and 75% seroconversion in H1N1 and H3N2 influenza strains, respectively, compared with 63% and 10% for aqueous-cream and intradermal influenza vaccination, respectively^12^. Several studies have demonstrated that imiquimod administered by intramuscular injection can be used as a vaccine adjuvant, but it is not known whether imiquimod can be used to treat live viral infections when administered intranasally. We therefore aimed to determine if TLR7 agonists provide protection against IAV-induced inflammation, lung dysfunction and morbidity in a mouse model.

Here, we demonstrate that delivery of imiquimod directly to the lungs *via* intranasal administration resulted in a reduction in viral replication, bodyweight loss, airway inflammation, neutrophil and eosinophil infiltration and pro-inflammatory cytokine expression following influenza A virus infection. Moreover, treatment with imiquimod substantially reduced pulmonary inflammation, improved several parameters of lung function including respiratory system resistance and increased several protective antibody isotypes. Collectively, these observations demonstrate TLR7 agonists are promising therapeutics in combating influenza virus pathology.

## Results

### Imiquimod and Aldara suppressed IAV-associated weight loss

**Figure 1:**
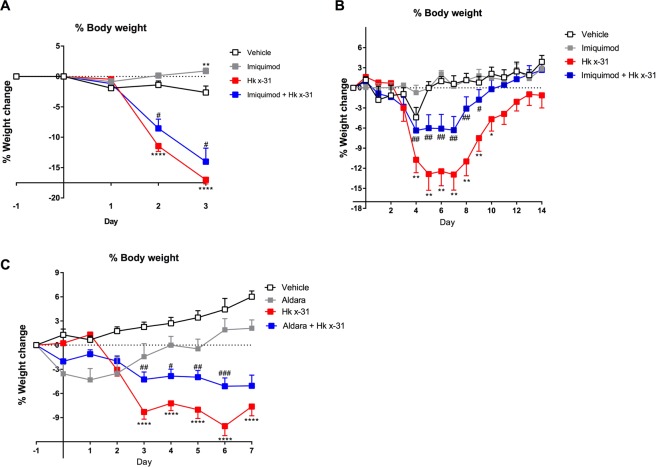
Effects of imiquimod and aldara on body weight loss induced by IAV. Naïve mice (vehicle; PBS) or mice infected with Hk x-31 at (**A**) 10^5^ PFU/mouse or (**B**) 10^3^ PFU/mouse, with daily intranasal treatments of either imiquimod (50 µg) or vehicle (PBS: DMSO; 1%), starting 1 day prior to infection up until one day prior to cull. Alternatively, in (**C**), mice were treated once daily with aldara (25 mg) or control on the back of the ear via epicutaneous application and infected with 10^4^ PFU/mouse of Hk x-31 or control (PBS). Data are represented as mean ± SEM of n = 7–13 mice. Data are expressed as % in body weight change from day −1, with statistical analysis conducted via a two-way ANOVA followed by Tukey’s *post hoc* test for multiple comparisons. *, **, and **** indicates p < 0.05, p < 0.01, and p < 0.0001 against vehicle control, respectively. #, ##, and ### indicates p < 0.05, p < 0.01 and p < 0.001 against Hk x-31, respectively.

Body weight was used as a surrogate marker of IAV-associated disease severity in mice. Starting from day 2, IAV-infected mice (10^5^ PFUs) began to lose significant body weight, reaching ~17% by day 3 (p < 0.0001 compared to vehicle control at equivalent time point). Imiquimod treatment significantly prevented IAV-induced weight loss, with mice losing ~13% of the initial bodyweight (p < 0.05) starting from day 2 (Fig. 1A).

At 10^3^ PFU of IAV, mice began to rapidly lose weight by day 3, reaching a maximum of ~15% at day 5, at which point it remained stable until day 8 where they began to recover and then fully recover by day 13 (p < 0.01) (Fig. 1B). Imiquimod treatment prevented this body weight loss, reaching a maximum of only ~6% by day 4. Mice fully recovered by day 10, which was 3 days earlier than the control group (p < 0.01, Fig. 1B). Imiquimod treatment of naïve mice resulted in either a modest ~2% reduction in bodyweight vs the vehicle control in Fig. 1A (p < 0.01) or had no effect on bodyweight (Fig. 1B).

Aldara treatment caused a significant reduction in bodyweight loss 3 days post-infection with 10^4^ PFUs of Hk x-31 IAV (Fig. 1C). Despite halting Aldara treatment at day 2 post-infection, Aldara treatment was still able to consistently reduce bodyweight loss from days 3 to 6 post-infection by ~50% compared to the virus alone cohort (Fig. 1C). Aldara treatment in naïve animals did not appear to cause any significant alterations in bodyweight, with a slight reduction in bodyweight initially (from day 0–2) but a full recovery was made after the cessation of treatment (Fig. 1C).

### Imiquimod suppressed IAV-induced airway inflammation, neutrophil and eosinophil infiltration

**Figure 2:**
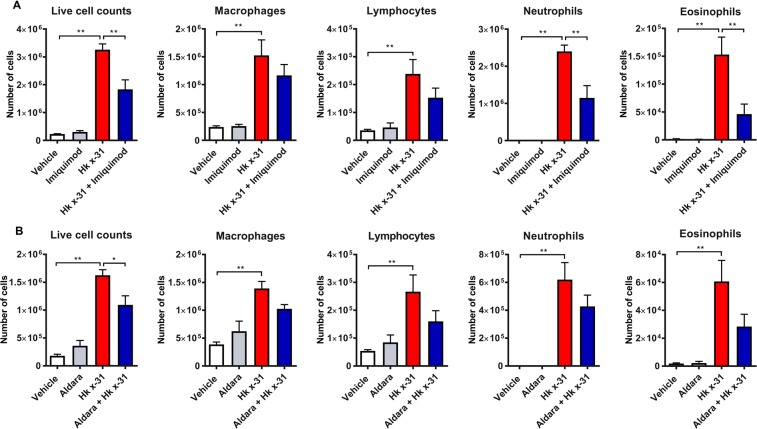
Imiquimod reduces airway inflammation and neutrophil infiltration in mice infected with IAV. Naïve mice (vehicle; PBS) or mice infected with Hk x-31 at (**A**) 10^5^ PFU/mouse and treated once daily with either imiquimod (50 µg) or vehicle (PBS: DMSO; 1%) via intranasal inhalation, starting 1 day prior to infection up until day 2 with analysis occurring at day 3. Alternatively, in (**B**), mice were treated once daily with aldara (25 mg) or control on the back of the ear via epicutaneous application and infected with 10^4^ PFU/mouse of Hk x-31 or control (PBS), where end point measurements were taken at day 3 post-infection. The total number of live cells and differential cell counts of macrophages, neutrophils, lymphocytes and eosinophils were measured. ~500 cells were counted from random fields by standard morphological criteria. Data are represented as mean ± SEM of n = 8–15 mice. Data are expressed as number of live inflammatory cells present in the BALF, with statistical analysis conducted via a one-way ANOVA followed by Tukey’s *post hoc* test for multiple comparisons. * and ** indicates p < 0.05, p < 0.01 and p < 0.0001, respectively.

Uncontrolled lung inflammation exacerbates the pathology caused by IAV infection. Live cells in the BALF were therefore taken to determine levels of airway inflammation. At day 3 (10^5^ PFU/mouse), IAV infection resulted in a substantial increase in airway inflammation (p < 0.0001, Fig. 2A,B). At day 3, imiquimod treatment suppressed airway inflammation caused by IAV infection by about 40% (p < 0.01, Fig. 2A).

Since imiquimod treatment reduced airway inflammation, we then proceeded to determine whether imiquimod treatment influenced the infiltration of specific cell populations within the BALF. At day 3 post infection (10^5^ PFUs), we observed extensive infiltration of macrophages (p < 0.001), lymphocytes (p < 0.05), neutrophils (p < 0.0001) and eosinophils (p < 0.0001) compared with the uninfected controls, reflective of airway inflammation (Fig. 2A). The predominant cell type in the airways at day 3 post infection was clearly neutrophils (Fig. 2A). Imiquimod treatment did not cause significant changes in macrophage or lymphocyte counts, but there was a significant ~50% reduction in neutrophil counts and ~70% reduction in eosinophil counts (p < 0.01; Fig. 2A).

We examined the impact of Aldara treatment on lung inflammation following IAV infection. Mice infected with IAV (10^4^ PFUs) displayed a significant increase in airway inflammation compared to the uninfected controls at 3 days post-infection (Fig. 2B). As expected, IAV infection caused a significant increase in the infiltration of macrophages, neutrophils, lymphocytes and eosinophils. Treatment with Aldara did not significantly reduce the numbers of these different inflammatory cell populations in the BALF, although there were trends for a reduction (Fig. 2B). Aldara treatment of naïve animals did not alter airway inflammation or change the cell populations (Fig. 2B). Overall, it appears that direct lung delivery of imiquimod *via* the intranasal route provides a stronger inhibitory effect against lung inflammation to IAV infection compared to delivery of the drug *via* the epicutaneous route.

### Imiquimod suppressed IAV-induced lung inflammation

**Figure 3:**
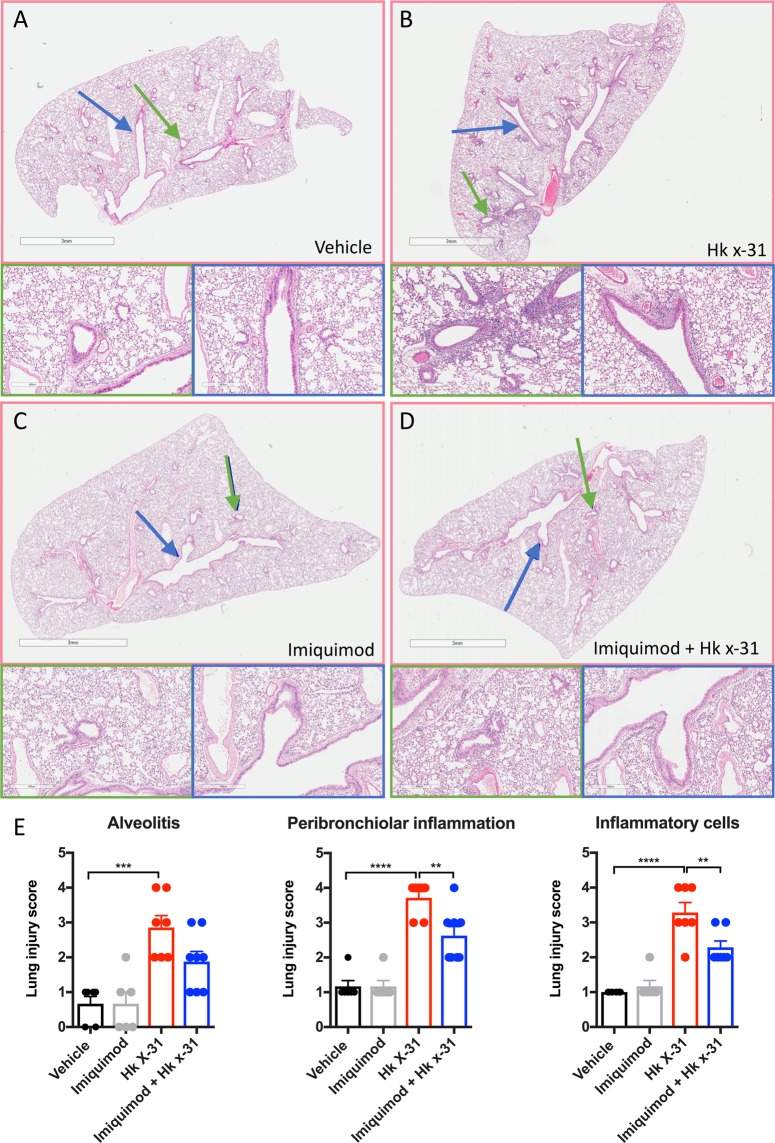
Lung histopathological stains reveal that imiquimod ameliorates IAV-induced lung injury. Haematoxylin and eosin stained paraffin lung sections from naïve mice (vehicle; PBS) treated with (**A**) PBS or (**B**) Imiquimod or mice infected with Hk x-31 at 10^5^ PFU/mouse, with daily intranasal treatments of either (**C**) PBS or (**D**) imiquimod (50 µg) starting 1 day prior to infection up until day 2 with analysis occurring at day 3 post-infection. Green arrow indicates small airway; blue arrow indicates large airway, where n = 6–8. Scale bars represent 3 mm in the overview and 400 µm for the zoomed images. (**E**) Each sample was scored blindly from 0–5 for each individual mouse (higher numbers indicate increased disease severity) from two independent assessors. Sections were scored for alveolitis, inflammatory cell infiltrate and peribronchiolar inflammation, with statistical analysis conducted via a one-way ANOVA followed by Tukey’s *post hoc* test for multiple comparisons. **, *** and **** indicates p < 0.01, p < 0.001 and p < 0.0001, respectively.

We next assessed the effect of imiquimod on lung inflammation and pathology at day 3 post-infection using H and E staining. Imiquimod treatment alone did not cause any discernible level of inflammation when compared with the vehicle controls (Fig. 3). IAV infection resulted in a drastic intensification in peri-bronchiolar inflammation, inflammatory cell infiltration and some degree of alveolitis (Fig. 3). Imiquimod treatment significantly suppressed IAV-induced peri-bronchiolar inflammation and inflammatory cell infiltration, with a strong trend for reduction in alveolitis. (Fig. 3).

### Effect of imiquimod on the NOX2 oxidase phagosomal oxidative burst

**Figure 4:**
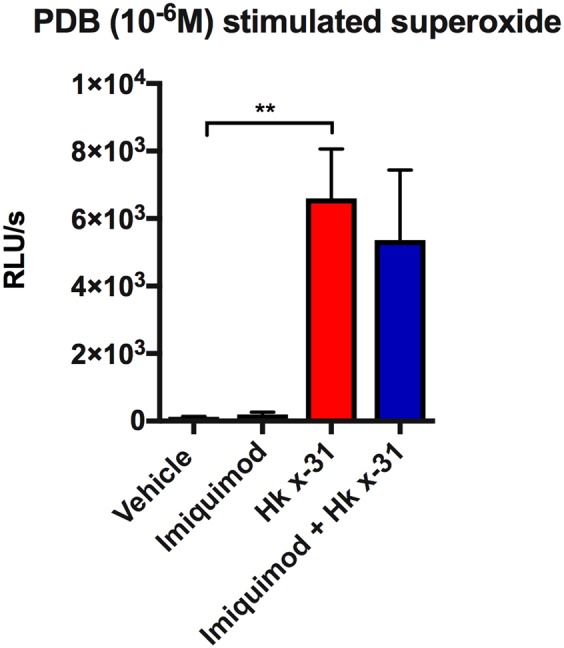
Effect of imiquimod on the oxidative burst in mice infected with Hk x-31 IAV. Oxidative burst measured by L-012 enhanced chemiluminescence in naïve mice (control; PBS) or mice infected with Hk x-31 at 10^5^ PFU/mouse with daily intranasal treatments of either imiquimod (50 µg) or vehicle control (PBS: DMSO; 1%), starting 1 day prior to infection up until day 2 with analysis occurring at day 3. Data are represented as mean ± SEM of n = 8–15 mice. Data are expressed as relative light units (RLU), measured as an average of triplicates over a 60-cycle period, subtracted by blank readings. Statistical analysis was conducted via a one-way ANOVA followed by Tukey’s *post hoc* test for multiple comparisons. ** indicates p < 0.01.

We examined whether imiquimod modifies reactive oxygen species (ROS) generation within the inflammatory cells present in the BALF. We stimulated cells with phorbol dibutyrate (PDB) to activate the oxidative burst, which is attributed to the nicotinamide adenine dinucleotide phosphate (NADPH) oxidase (NOX2) NADPH oxidase enzyme. BALF inflammatory cells taken from IAV infected mice possessed a ~70-fold increase in the oxidative burst at day 3 (10^5^ PFUs) compared to cells from uninfected controls (p < 0.01, Fig. 4). Imiquimod treatment did not modify this BALF inflammatory cell ROS production (Fig. 4).

### Imiquimod suppressed influenza titers and neutrophil chemotactic cytokines

**Figure 5:**
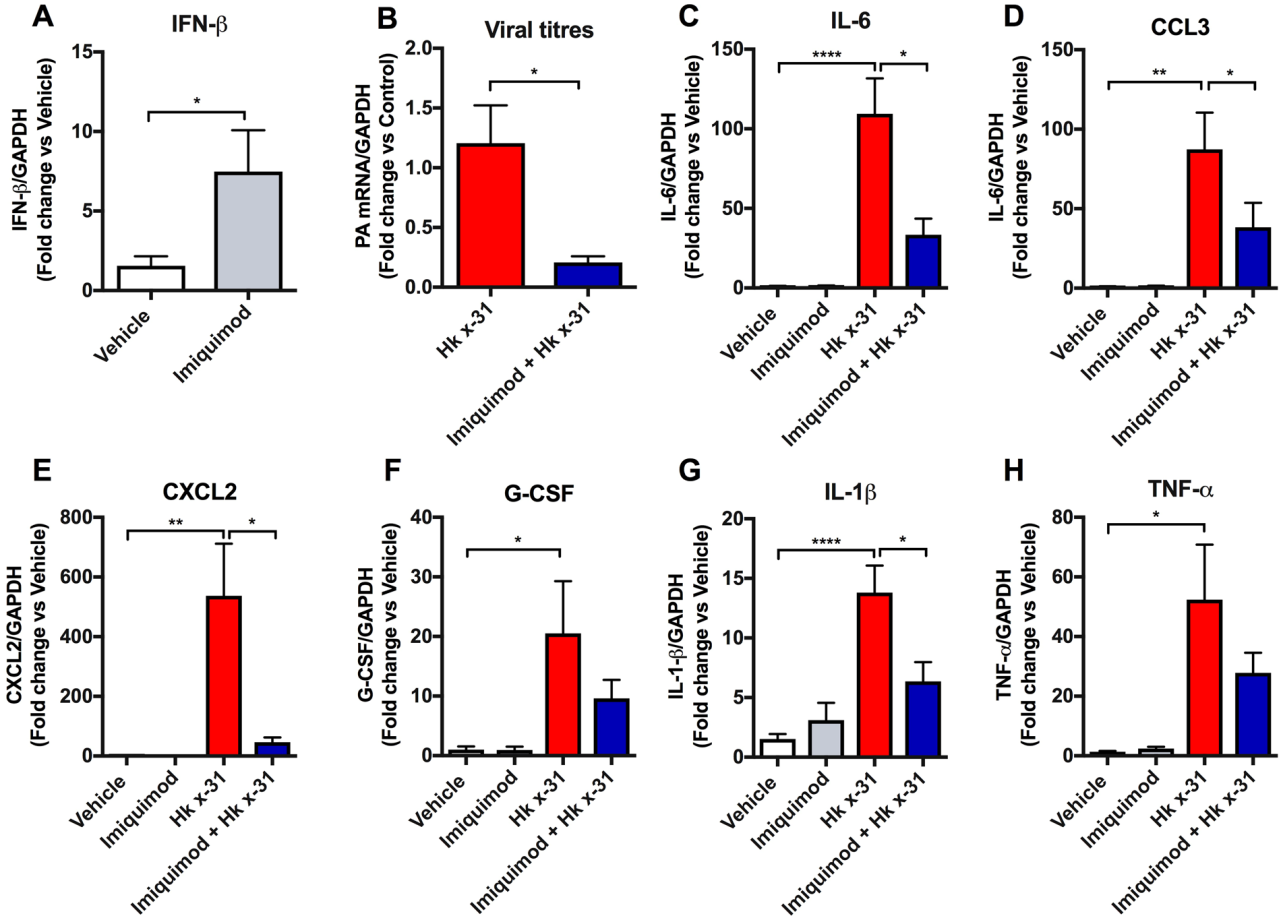
Effects of imiquimod on Type I IFN, viral load and IAV-induced cytokine mRNA expression. Cytokine and influenza A virus polymerase (PA) mRNA expression measured with qPCR in naïve (control; PBS) or mice infected with Hk x-31 at 10^5^ PFU/mouse with daily intranasal treatments of either imiquimod (50 µg) or vehicle (PBS: DMSO; 1%) control, starting 1 day prior to infection up until day 2 with analysis occurring at day 3. Data are represented as mean ± SEM of n = 6–16 mice. Responses are relative to GAPDH and then expressed as a fold-change above vehicle controls. Statistical analysis conducted via a one-way ANOVA followed by Tukey’s *post hoc* test for multiple comparisons for C-H), or an unpaired student’s T test for (**A**,**B**). *, ** and **** indicates p < 0.05, p < 0.01 and p < 0.0001, respectively.

It is well established that TLR7 activation by imiquimod drives a powerful anti-viral cytokine program characterized by elevated levels of Type I IFNs. We confirmed this by showing that intranasal imiquimod treatment results in a significant ~5-fold elevation in Type I IFN-β in the lung tissue of mice following 3 days of treatment (p < 0.05, Fig. 5A). We examined the impact of imiquimod treatment on influenza virus replication by assessing mRNA expression of the influenza polymerase gene (PA) by qPCR. We chose to assess this at Day 3 post infection, which represents the peak of the inflammatory response to influenza infection in mice models. Imiquimod treatment significantly reduced PA mRNA expression in the lung tissue compared to the untreated control influenza infected mice (p < 0.05, Fig. 5B).

Thus far we have shown that imiquimod treatment selectively reduced neutrophil infiltration in the lungs following influenza A virus infection. Cytokines play a key role in regulating the innate immune system against pathogens including IAV. Therefore, using qPCR, we determined the expression of a variety of cytokines with neutrophil chemotactic properties including IL-6, CCL3, CXCL2 and G-CSF, as well as pro-inflammatory cytokines IL-1β and TNF-α at day 3 (10^5^ PFUs) post-infection. Naïve mice treated with imiquimod had no significant increases in cytokine mRNA expression compared with the vehicle control (Fig. 5C–H). IAV infection increased the mRNA expression at day 3 post-infection (10^5^ PFUs) of IL-6, CCL3, CXCL2, and G-CSF, IL-1β and TNF-α (p < 0.05, Fig. 5C–H). Imiquimod treatment significantly suppressed the mRNA levels of IL-6, CCL3, CXCL2 and IL-1β (p < 0.05), but had no effect on G-CSF or TNF-α, although there was a strong trend for a reduction (Fig. 5C–H).

### Imiquimod improves lung function in IAV infected mice

**Figure 6:**
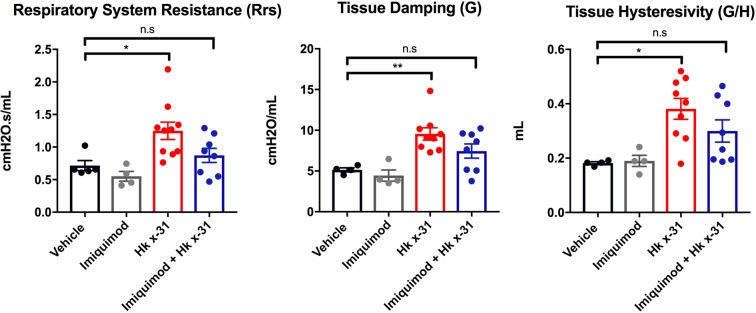
Imiquimod causes an improvement in respiratory system resistance, tissue damping and tissue hysteresivity. Naïve (control; PBS) or mice infected with Hk x-31 at 10^5^ PFU/mouse were treated once daily via intranasal administration of either imiquimod (50 µg) or vehicle control (PBS) starting 1 day prior to infection up until two days post-infection with analysis occurring at day 3. Mice were anaesthetized and connected to the flexiVent machine and mechanically ventilated at 300 breaths per minute. Lung function measurements were recorded on the fliexiVent machine for respiratory system resistance, tissue damping and tissue hysteresivity. Data are represented as mean ± SEM of n = 4–9 mice. The average of triplicates were taken, where statistical analysis was conducted via a one-way ANOVA followed by Tukey’s *post hoc* test for multiple comparisons. n.s. denotes not significant, * and ** indicates p < 0.05 and p < 0.01, respectively.

Acute infection with influenza A virus can cause impairment in lung function due to cellular inflammation arising from an excessive immune response. Imiquimod treatment of naïve animals did not change any of the baseline parameters (Fig. 6A–C). In accordance with^13^, influenza A virus infection caused a significant elevation in respiratory system resistance of the conducting airways that measures the degree of constriction in the lungs, tissue damping (the amount of energy dissipated from the alveoli) and tissue hysteresivity (elastic stiffness within the respiratory system over the degree of energy dissipated). However, influenza A virus infection in mice treated with imiquimod displayed no significant increases in respiratory system resistance, tissue damping and tissue hysteresivity (Fig. 6A–C). Thus, imiquimod protects against lung dysfunction caused by influenza A virus infection.

### Imiquimod induced a more potent local antibody response in the lungs

**Figure 7:**
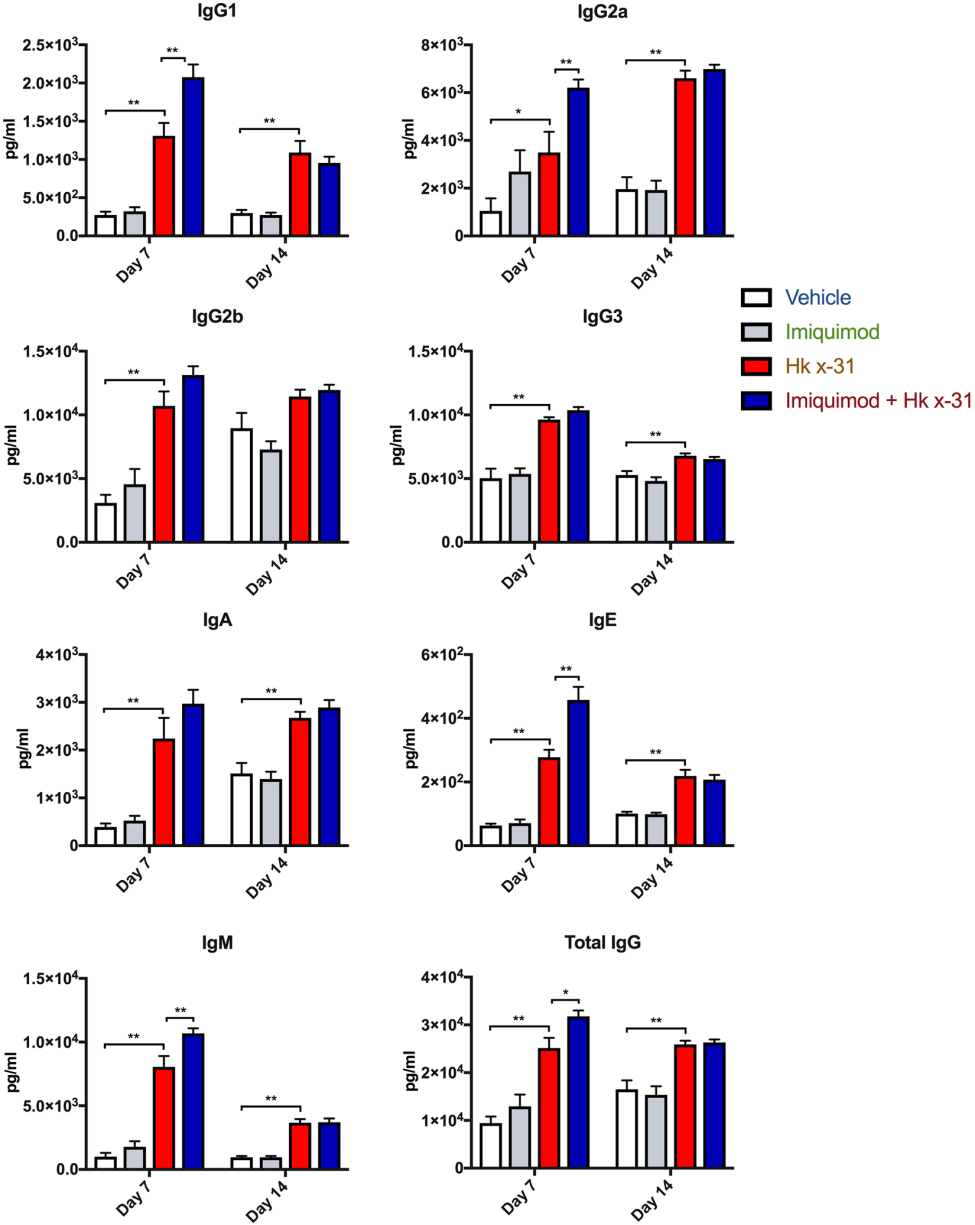
Effects of imiquimod on IAV-induced antibody titers in BALF. Antibody titers measured by multiplex antibody assays in naïve (control; PBS) or mice infected with Hk x-31 at 10^3^ PFU/mouse, with daily intranasal treatments of imiquimod (50 µg) or vehicle control (PBS: DMSO; 1%), starting 1 day prior to infection up until day 2 with analysis occurring either at day 7 or day 14. Data are represented as mean ± SEM of n = 5–13 experiments. Data are expressed as picograms per milliliter (pg/ml), with statistical analysis conducted via a one-way ANOVA followed by Tukey’s *post hoc* test for multiple comparisons. * and ** indicates p < 0.05 and p < 0.01, respectively.

We next determined whether imiquimod treatment influences antibody production following influenza A virus infection. To perform these studies mice were infected with a low non-lethal dose of influenza i.e. 10^3^ PFU/mouse and both serum and BALF antibody production were determined at Day 7 and Day 14 post infection by use of a multiplex assay kit. At day 7, IAV infection caused significant increases in IgG1 (~6.5-fold increase, p < 0.01), IgG2a (~1.8 fold increase, p < 0.01), IgG2b (~3-fold increase, p < 0.01), IgG3 (~2-fold increase, p < 0.0001), IgA (~5-fold increase, p < 0.01), IgE (~4.5-fold increase, p < 0.01), IgM (~8-fold increase, p < 0.0001), and total IgG (~3-fold increase, p < 0.001) production (Fig. 7) compared to the naïve vehicle controls. Imiquimod treatment yielded a significantly higher antibody response in IgG1 (~1.8-fold, p < 0.05), IgG2a (~2-fold, p < 0.05), IgE (~1.6-fold, p < 0.01) and IgM (~1.25-fold, p < 0.05) during IAV infection (Fig. 7).

At Day 14, IAV infection caused increases in IgG1 (~3 fold, p < 0.0001), IgG2a (~3-fold, p < 0.0001), IgG3 (~1.25-fold, p < 0.001), total IgG (~1.5-fold increase, p < 0.0001) and IgE (~2-fold, p < 0.0001) within the BALF (Fig. 7). Unlike at day 7, imiquimod treatment did not result in any further increases in antibody response at day 14 (Fig. 7).

**Figure 8:**
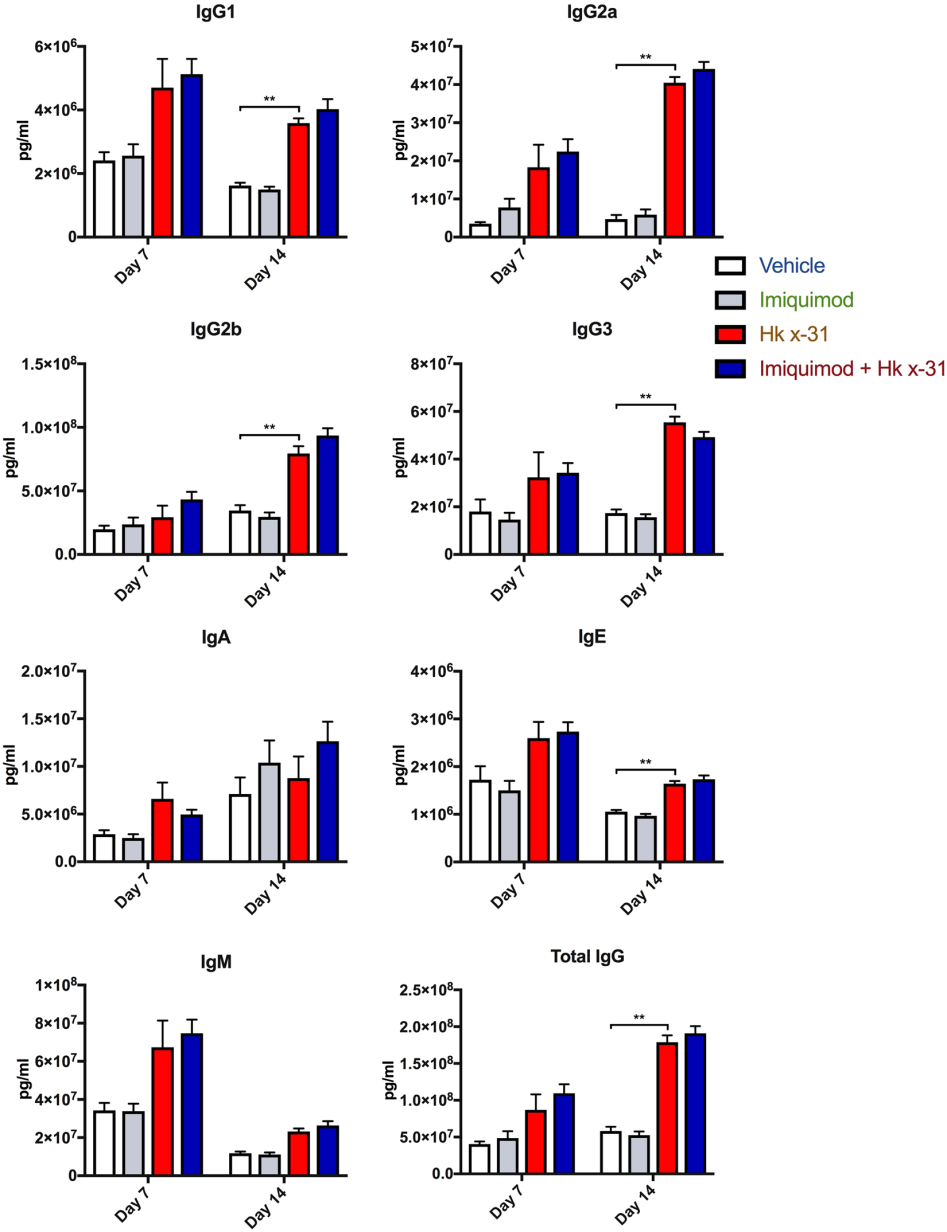
Imiquimod does not alter antibody titers in serum. Antibody titers measured by multiplex antibody assays in naïve (control; PBS) or mice infected with Hk x-31 at 10^3^ PFU/mouse, with daily intranasal treatments of imiquimod (50 µg) or vehicle control (PBS: DMSO; 1%), starting 1 day prior to infection up until day 2 with analysis occurring either at day 7 or day 14. Data are represented as mean ± SEM of n = 5–13 experiments. Data are expressed as picograms per milliliter (pg/ml), with statistical analysis conducted via a one-way ANOVA followed by Tukey’s *post hoc* test for multiple comparisons. ** indicates p < 0.01.

In the serum, IAV infection caused no significant alterations in all the Ig production at day 7 post infection. Day 14 serum data showed significant increases in IgG1 (~2-fold, p < 0.001), IgG2a (~10-fold, p < 0.0001), IgG2b (~2-fold, p < 0.0001), IgG3 (~3.2-fold, p < 0.001), IgE (~1.6-fold, p < 0.001) and total IgG (~3-fold, p < 0.0001) with no effects on IgA and IgM in IAV compared to uninfected controls (Fig. 8). However, imiquimod treatment did not further enhance Ig production during IAV infection in the serum at either day 7 or 14 (Fig. 8).

## Discussion

Influenza A virus infection causes significant pathology, which is attributed to an over-exuberant inflammatory response in the airways and lungs. At the acute phase of infection, this is largely characterized by a substantial infiltration of neutrophils and other inflammatory cells including macrophages and monocytes in the airways resulting in oxidative stress and pro-inflammatory cytokine release. Although the influenza A virus is eventually cleared and the inflammation resolved, there is significant morbidity and in many cases, if this over-exuberant inflammatory response goes too far, it can result in death from primary pneumonia. Intramuscular administration of TLR ligands has displayed protective outcomes against influenza virus infections^7^. However, the studies mentioned above do not address the effects of these ligands on the inflammatory responses and lung injury induced by influenza viruses. In the present study, we demonstrate unequivocally that prophylactic imiquimod administration alleviates the morbidity caused by influenza infection by re-programming the inflammatory response. We show that imiquimod not only dampens the acute inflammatory response, primarily *via* suppression of neutrophil recruitment, but it improves viral clearance and disease resolution.

Prophylactic administration of conjugated TLR7 ligands have been shown to be protective against IAV-induced lethality^14,15^. Due to ethical restraints, we were unable to explore whether imiquimod protects against mortality. The reduction in disease severity as measured by body weight loss, however, seems to be indicative of a reduction in IAV-induced lethality. Body weight can be used as a surrogate marker of disease severity following IAV infections. In the present study, we demonstrated that mice infected with influenza A virus displayed a significant weight loss that was highly dependent on the initial dose of the virus. Imiquimod treatment significantly prevented body weight loss across all doses of virus used and this effect was most pronounced for the low dose viral infection model. Imiquimod markedly diminished the weight loss caused by low dose IAV (10^3^ PFUs), with a full recovery occurring approximately 3 days earlier than the control group. Consistent with this, treatment with Aldara also prevented bodyweight loss by ~50%. This maintenance of body weight could be due to imiquimod combatting infection by priming the immune response, thus there is an earlier and more efficient response particularly at lower infection doses. Indeed, we found that imiquimod treatment resulted in a significant reduction in the peak viral burden in the lungs at Day 3 post infection compared to the untreated control mice. This is likely to be attributed to the well-established Type I IFN response triggered by TLR7 activation by imiquimod. TLR7 activation has previously been shown to inhibit viral growth and infection *in vitro* using chicken macrophages^16^. In addition, viral titers were significantly reduced in a rat model of influenza when prophylactically given a dual TLR7/TLR8 agonist^17^. In this study, we propose that the enhanced capacity of the innate immune system to clear virus during the acute infection phase has a profound impact on the disease pathogenesis including weight loss, lung inflammation and lung function and is attributed to the specific activation of TLR7.

Inflammation is an integral part of the host immune process that is normally associated with the clearance of invading pathogens. However, excessive inflammation as a consequence of IAV infection, causes significant collateral damage, and particularly lung pathogenesis and cell necrosis. We therefore addressed whether imiquimod influences airway inflammation by analysing live cell counts within the BALF of viral infected mice. IAV caused significant infiltration of leukocytes into the lungs of infected mice, which was consistent with previous studies^18,19^. Imiquimod treatment significantly reduced the airway inflammation caused by IAV infection and this is likely to underpin the decrease in morbidity that we observed. We also determined whether or not imiquimod modified specific sub-populations of inflammatory cells within the BALF: namely macrophages, lymphocytes, neutrophils and eosinophils. Consistent with our previous data^18,19^, IAV infection caused significant increases in all four of these inflammatory cell populations and particularly in neutrophils, with little to no presence in naïve mice, to representing the most prominent cell type during IAV infection. Imiquimod suppressed both neutrophil and eosinophil cell counts. In order for the virus to be completely cleared at the site of infection, an important arm of the adaptive immune response is activated resulting in a cytotoxic T lymphocyte (CD8^+^; cluster of differentiation) and T helper cell (CD4^+^) response^20–22^. The lack of effect of imiquimod treatment on overall lymphocyte numbers, suggests that TLR7 activation *via* intranasal imiquimod delivery preserves this critical arm of the adaptive immune system and thus allowing for effective viral clearance.

Alveolar macrophages are essential for protection from respiratory failure and associated morbidity following influenza A virus infection^23,24^. Infection of alveolar macrophages is an early event in recognition of the virus by the innate immune system and although these cells are susceptible to influenza virus infection and viral proteins are produced, replication is abortive and infectious progeny are not released. During influenza exposure, alveolar macrophages release proinflammatory cytokines and Type I IFNs, which limit viral replication and spread within the respiratory tract. However, the cells will also die by apoptosis and then are replenished by the infiltration of blood borne monocytes. In the present study we show that influenza infection results in a significant increase in airway macrophages at Days 3 and 5, which is a typical response to this virus^25^. There are typically three subpopulations of these macrophages at these phases of infection including resident alveolar macrophages, interstitial macrophages and monocytes. Importantly, imiquimod treatment did not modify overall macrophage numbers in the airways and lungs and this effect is critical as it would suggest that macrophage function is preserved. It is likely that these macrophages are polarized towards an anti-inflammatory M2 phenotype as it is consistent with the reduction in viral burden and are more rapid resolution phase. We also established the key mediators that drive neutrophil recruitment to the airways and determined how imiquimod abrogates this response. We focused on several key chemotactic factors that are involved in the migration and trafficking of neutrophils including IL-6, CCL3, CXCL2, G-CSF and IL-1β^26–28^. We demonstrated that imiquimod caused a significant reduction in mRNA expression of a number of these neutrophil chemotactic cytokines in lung tissue. It remains to be established how TLR7 activation by imiquimod suppresses neutrophil chemotactic cytokines. One possibility lies in the production of Type I IFNs by imiquimod that have been shown to suppress the production of certain neutrophil chemotactic cytokines such as CCL2 and MIP-2^29^.

Although neutrophils can potentially have both protective and pathogenic roles following influenza A virus infections, we propose that the protective properties of imiquimod are due to the partial suppression of neutrophilia in the airways and lungs. We postulate that a critical part of lung pathogenesis due to influenza infection is governed by the degree of neutrophil migration into the lungs. Importantly, >90% depletion of neutrophil populations through the use of monoclonal antibodies (mAb, RB6–8C5 and 1A8) results in significantly worse outcomes for mice infected with IAV using the Hk x-31 strain^30,31^, including more significant weight loss. This is not surprising, given that neutrophils are capable of minimizing viral burden. In contrast suppression of MIP-2 with a monoclonal antibody, which reduced neutrophil numbers to about 40–50% of control, resulted in a significant protective effect against influenza infection. Moreover, antagonism of CXCR1/2 has previously been shown to reduce neutrophil infiltration that was associated with marked improvements in lung function, a reduction in pulmonary inflammation and lethality rates in IAV-infected mice^32,33^. Again, with these studies, there was a partial reduction of neutrophilia. This suggests that the dynamic balance between inflammation and cellular infiltration is critical for limiting viral pathogenesis and this is consistent with our findings showing that a ~40–50% reduction in neutrophil numbers is associated with reduced morbidity. How neutrophils contribute to this morbidity is not really known, but it could possibly involve several mechanisms including excess protease and extracellular trap release leading to lung injury, and primary pneumonia^34^. Indeed, reduction of neutrophils could explain the improved lung pathology when mice where treated with imiquimod, which would be consistent with reduced degranulation and byproducts that can harm the host. Thus, ameliorating excessive neutrophil inflammation in the lung is a possible explanation for the effect of imiquimod on viral pathogenesis.

Generally, eosinophils are associated with allergic reactions, including chronic asthma^35^, however eosinophils can also play a protective role within influenza infections. Retrospective studies on the 2009 swine flu pandemic found that although asthmatics were more likely to be hospitalised due to IAV-induced asthmatic attacks^36^, allergic inflammation was protective against severe IAV-associated morbidity and mortality^37^. Indeed, upon infection, eosinophils upregulate expression of the Major Histocompatibility Complex (MHC) class I and CD86 antigen presentation markers, leading to activation and enhancement of the CD8^+^ T cell response^38^. This suggests that there is a dynamic balance between effective lung infiltration and an excessive immune response that promotes pathogenesis. In our study, we found that imiquimod treatment reduced eosinophil infiltration in the BALF of IAV-infected mice and imiquimod has previously been shown to inhibit chronic inflammation in a mouse model of asthma^39^. More specifically, our study shows that TLR7 agonist has the potential to modulate viral pathogenesis and could be used to reduce hospitalisations in low pathogenic, IAV-induced asthmatic attacks without compromising host immunity.

We measured ROS production from BALF inflammatory cells using L-012-enhanced chemiluminescence. It is noteworthy that this method of ROS detection is specific for measuring ROS generation from NOX2 oxidase and provides a measure of the oxidative burst capacity of the cells *per se*, and not an overall marker of lung oxidative stress. In the present study, imiquimod had no effect on this oxidative burst capacity of BALF inflammatory cells, either in naïve controls or in IAV infection. Therefore, imiquimod does not modify the intrinsic capacity of inflammatory cells to produce ROS. However, the overall production of ROS and the ensuing lung oxidative stress is likely to be substantially less following imiquimod treatment because there was a marked reduction in overall numbers of inflammatory cells, in particular neutrophils, which generate the highest levels of ROS of all inflammatory cells. Lung oxidative stress has previously been associated with IAV virulence and disease and we have previously shown that a significant increase in lung oxidative stress occurred with IAV infection^19,40,41^ but ablating this by use of the NOX2^−/y^ mouse or with NOX2 oxidase specific inhibitors was associated with less pathology.

Dysregulated lung function is a hallmark feature that underpins the severity of influenza virus infections. Our study demonstrates infection with the low pathogenic virus caused a marked elevation in total respiratory system resistance (Rrs), likely due to mucus formation and the influx of inflammatory cells in the lungs. By performing low-frequency forced oscillation technique, we were able to demonstrate that there was no increase in central or conducting airways resistance (Rn), but rather tissue damping (G) and tissue hysteresivity were increased in virus-infected mice, which are parameters related to tissue or alveoli resistance. The increase in tissue resistance is likely to be driven by multiple pathological features including alveoli inflammation, shedding of epithelial cells and the accumulation of debris. Importantly, imiquimod treatment prevented an increase in total and tissue resistance, tissue damping (G) and tissue hysteresivity which were associated with its anti-inflammatory activity.

TLR7 and Type I IFNs have been shown to indirectly stimulate and activate B cells, hence we investigated whether imiquimod treatment would lead to an enhanced antibody response^42^. We examined 7 different isotypes both locally (BALF) and in the periphery (serum). Viral infection resulted in an increase in all subtypes examined including IgA, IgE, IgG1, IgG2a, IgG2b, IgG3 and IgM within the BALF, however there seemed to be a relative lack of response within the serum at day 7 (Fig. 7). At day 14 however, we observed significant increases in serum antibody levels in response to IAV infection. These differences suggest an initial, localized adaptive immune response before it is spread systemically. Importantly, following imiquimod treatment, there was a further significant enhancement in BALF IgG1, IgG2a, IgE, IgM and total levels of IgG. We showed a failure of imiquimod to raise IgA in the BALF, which is a little surprising, as IgA is the primary antibody present in mucosal membranes of the upper airways and more specifically in the nasal passage^43^. It may therefore be possible that imiquimod treatment raised IgA antibody titres within the mucous in the upper respiratory tract, such as the nasal compartments, which warrants further investigation. We additionally observed strong increases in IgM production at day 7 in influenza infected mice, and IgM antibody production was further enhanced by imiquimod treatment. Conversely, we saw a rapid decline in IgM production at day 14, and this might be due to IgM undergoing a class switch to other isotypes^44^. IgE increased drastically by influenza infection, and further by imiquimod treatment at day 7. This is inconsistent with a previous study, as it described IgE as unaffected in influenza infection in the absence of an allergen^45^. However, there were differences in experimental protocol that must be addressed. Firstly, Suzuki *et al*. targeted specifically for anti-ovalbumin IgE antibodies, whilst we non-specifically measured IgE. Additionally, Suzuki *et al*. involved measurement at 22 days post infection^45^. These data seem to be more reflective of our 14 day IgE response, which indicates that either (a) there is a non-specific increase in IgE antibody generation early in infection, or (b) IgE plays an unknown role in IAV infection early in disease. Overall we provide evidence that imiquimod induces a faster antibody response, and therefore primes the immune system for an increased and more rapid capacity for viral neutralization, which may have aided in the faster recovery seen in the body weight. As there were no observable differences in the serum due to imiquimod treatment, we suggest that intranasal administration of imiquimod only affected the lower respiratory tract.

The immunopathological profile of influenza A virus infection in mice is generally characterized by a rapid infiltration of neutrophils and macrophages in the first 48–72 hrs. In the lung tissue, there is extensive peri-bronchial inflammation and oxidative stress and the viral burden has peaked and mice generally lose significant weight. Typically, this inflammatory response resolves in 7–10 days and there is a powerful adaptive cytotoxic T cell response that helps eventually eliminate the virus. In the present study, we have shown that the TLR7 agonist imiquimod significantly modifies the immune system response to influenza. The administration of the TLR7 agonist imiquimod *via* the intranasal route resulted in a dramatic reduction in the morbidity and airways and lung inflammation caused by influenza A virus infection. The reduction in inflammation was largely due to a suppression of neutrophil infiltration and there was a preservation of macrophage and T-lymphocyte infiltration. Therefore, imiquimod powerfully suppresses the over-exuberant innate inflammatory response to influenza without compromising critical arms of the adaptive immune system. Aldara is a prescription medication that is used to treat genital warts, superficial basal cell carcinoma, and actinic keratosis. Here we suggest of imiquimod for the treatment of influenza A virus infection via intranasal delivery. This latter mode of delivery provides a stronger priming of the immune response to influenza than epicutaneous delivery.

In summary, the present study builds upon a wealth of literature that places TLR7 as a critical sensor of viral infection, a powerful antiviral signaling system and thus a strong candidate target for the treatment of respiratory viral infections such as influenza infection.

## Materials and Methods

### Mice and animal ethics

8–12-week-old C57BL6/J male mice were obtained from either the Monash Animal Services (Monash University, Clayton, Australia) or the Animal Resources Centre, Australia (ARC) (Western Australia, Australia). Mice were housed in the animal research facilities at either Monash University, Clayton, Australia or RMIT University, Bundoora, Australia under standard conditions with non-restricted access to food and water. The animals used in these experiments were approved by both the Monash University Animal Research Platform Animal Ethics Committee (ethics number MARP/2016/024) and the RMIT Animal Ethics Committee (ethics number 1706). The experiments were conducted in compliance with the guidelines of the National Health and Medical Research Council (NHMRC) of Australia on animal experimentation.

### Influenza A Virus

Mouse-adapted Hong Kong X31 (Hk x-31; H3N2) was provided by Prof Patrick Reading (Department of Microbiology and Immunology, Doherty Institute, The University of Melbourne) at 6.7 × 10^8^ plaque forming units/ml (PFU/mL) and stored at −80 °C. Aliquots were thawed and diluted on the day of use with phosphate buffered saline (PBS; Sigma Aldrich, St Louis, USA, cat. no. D8537) when required for *in vivo* infections.

### Virus infection models and treatment regimens

Mice were treated once daily with either imiquimod (50 µg) *via* intranasal administration or Aldara (25 mg; 5% imiquimod) *via* epicutaneous application to one ear. Control mice were treated with the PBS vehicle. The treatment regimen started at day −1 until day 2 for studies involving a high (10^5^ PFUs/mouse) or low dose (10^3^ PFUs/mouse) Hk x-31 infection. In all experiments, Hk x-31 infections or PBS controls (50 µL) were started at Day 0. Intranasal imiquimod or vehicle controls occurred in anaesthetized mice (mixed isoflurane (5 L/min) and O_2_ (2 L/min) using the Stinger isoflurane machine; AAS medical, Sydney, Australia). Mice were culled either at day 3 (10^5^ PFU/mouse) or day 7 and 14 (10^3^ PFU/mouse) *via* intraperitoneal injection of 0.2 mL(v/v) ketamine/xylazine (Troy Laboratories Pty. Ltd., Glendenning, Australia) in PBS (final concentration of 11.1%). Body weights were recorded daily.

### Extraction of Bronchoalveolar lavage fluid and lung tissue

Bronchoalveolar lavage (BAL) was performed to extract the cells in the lung to assess inflammation. The mouse trachea was exposed, and a small incision made roughly three quarters the way up from the ribcage. A 19-gauge needle was inserted, and lungs were flushed with PBS; once with 0.4 mL followed by 3 × 0.3 mL. BALF was stored in Eppendorf tubes chilled on ice. Following lavage, the left lung was fixed using 10% neutral buffered formalin, and used for histological analysis. The right lung was separated into lobes, and snap frozen in liquid nitrogen. Lobes were then stored at −80 °C prior to PCR. The BALF obtained was used for subsequent determination of airway inflammation through total and live cell counts, and ROS production *via* L-012-enhanced chemiluminescence.

### Analysis of airway inflammation and cell differentials

A mixture of 10 μL of 0.4% Trypan Blue Stain (Invitrogen, Thermo Fisher Scientific, Carlsbad, USA, cat. no. T10202) and 10 μL BALF was analysed *via* the Countess Automated Cell counter (Invitrogen, Thermo Fisher Scientific, Carlsbad, USA) in duplicates and averages were taken to obtain the total and live cell counts. Alternatively, 10 μL of BALF was mixed with 10 µL ethidium bromide (10 mg/mL; Invitrogen, Thermo Fisher Scientific, Carlsbad, USA, cat. no. 15585011) in PBS (1%) and counted using a hemocytometer.

The remaining BALF was centrifuged at 3 × g for 5 mins. The supernatant was discarded, and the pellet was resuspended in pre-warmed (37 °C), sterile PBS at a final concentration of 250,000 cells/mL. 50,000 cells were then used to prepare cytospin slides (Cytospin 3, Shandon, UK) for differential cell counting as previously published^18^. Cells were differentiated into macrophages, neutrophils, eosinophils and lymphocytes using standard morphological criteria and at least 500 cells/slide were counted from random fields.

### Superoxide detection with L-012-enhanced chemiluminescence

L-012 is a luminol-based probe that is a commonly used to detect the generation of NOX-derived superoxide. The probe reacts with different types of ROS, including superoxide anions to produce luminescence at long wavelengths^46,47^. 50,000 cells from each BALF sample were seeded in triplicates onto a 96-well Opti Viewplate with Dulbecco’s Modified Eagle Medium (DMEM; Sigma Aldrich, St Louis, USA, cat. no. D6046) in 10% (v/v) fetal bovine serum (Sigma-Aldrich, St Louis, USA, cat. no. 12003 C) added to make a total volume of 200 μL per well. Cells were washed with warm 37 °C Krebs-HEPES buffer and exposed to a Krebs-HEPES buffer containing L-012 (10^−4^ mol/L) in the absence (i.e. basal ROS production) or presence (stimulated ROS production) of the protein kinase C (PKC) and NADPH oxidase activator phorbol dibutyrate (PDB; 10^−6^ mol/L). The same treatments were performed in blank wells (i.e. with no cells), which served as controls for background luminescence. All treatment groups were performed in triplicates. Photon emission [relative light units (RLU)/s] was detected using the Chameleon™ luminescence detector (Hidex, model 425105, Finland) and recorded from each well for 1 s over 60 cycles. Individual data points for each group were derived from the average values of the three replicates minus the respective blank controls.

### Analysis of cytokine expression using Real-time qPCR

Whole lungs were perfused free of blood via right ventricular perfusion with 10 mL of pre-warmed saline, rapidly excised en-bloc, blotted and snap frozen in liquid nitrogen. Total RNA was extracted using RNeasy kits (Qiagen), reverse transcribed with SuperScript III (Invitrogen), and triplicate real time PCR reactions were performed with Applied Biosystems pre-developed assay reagents, as previously described^18^. Universal PCR Master Mix (Applied Biosystems, Foster City, CA, USA) or SYBR Green PCR Master Mix (Applied Biosystems, Foster City, CA, USA) and analyzed on ABI StepOneTM and StepOnePlusTM Real-time PCR Systems (Perkin-Elmer Applied Biosystems, Foster City, CA, USA). The PCR primers for TNF-α, IL-1β, IFN-β, IL-6, CCL3, CSF3, CXCL2 and IL-17A were included in the Assayon-Demand Gene Expression Assay Mix (Applied Biosystems, Foster City, CA, USA). Additionally, a custom designed forward and reverse primer of the segment 3 polymerase (PA) of influenza virus was used to measure viral titres. Data are presented relative to the GAPDH mRNA and normalised to the naive control.

### Histological examination of lung sections via H&E staining

As described above, the left lung was dissected from mice and fixed in neutral buffered formalin (10%) for 24–48 hours. Lungs were then processed in paraffin wax, and sections were cut between 3–4 μm thick longitudinally. Sections were then stained with hematoxylin and eosin (H&E). Slides were scanned by light microscopy and uploaded to Aperio microscope scanner (Leica biosystems, Nussloch, Germany). Histology was performed by the Department of Histology (Monash University, Clayton, Australia), and analysed using an Aperio Imagescope. The lung injury score was determined by a grading system that combined assessments of alveolitis, inflammatory cell infiltration and peribronchiolar inflammation by two independent assessors based on previous literature^30^. A score of 0 was indicative of healthy lungs (i.e. no damage); 1-very mild damage; 2-mild damage; 3-moderate damage, 4-severe damage and 5-extremely severe histological changes.

### Assessment of lung function

Baseline lung function measurements were carried out using a low-frequency forced oscillation technique (LFOT) and a small-animal ventilator (flexiVent; Scireq, Montreal, QC, Canada) as previously published^13^. Mice were anaesthetized with a mixture of ketamine and xylazene (100 mg/kg and 20 mg/kg) via *i.p*. injection. A small incision was made to the trachea, allowing for insertion of a cannula that was secured using silk thread. Mice were then connected to the flexiVent and ventilated at 300 breaths per minute and single/low-frequency forced oscillation techniques were performed to measure and partition respiratory system resistance (Rsr), tissue damping (G) and tissue hysteresivity (tissue damping/tissue elastance), where the average of triplicates for each parameter were used.

### Measurement of antibody levels in serum and BALF

To obtain serum, a cardiac puncture was performed to get up to 1 mL of deoxygenated blood. Blood was then centrifuged at 4 °C at 200 × g for 5 mins and the serum aspirated and aliquoted into 2 Eppendorf tubes, containing ~100 μl of serum each. Serum was then snap frozen and stored at −80 °C until required for measurement of antibody titer. For BALF, a bronchoalveolar lavage was performed as described above. BALF was then centrifuged at 3 × g for 5 mins. The pellet was discarded, and BALF was snap frozen and then stored at −80 °C.

Antibody assay was performed on serum and BALF using the Mouse Isotyping 7 plex ELISA Kit (Affymetrix eBioscience, California, USA, cat. no. EPX070-20815-901), measuring the antibody isotypes IgA, IgE, IgG1, IgG2a, IgG2b, IgG3, IgM and total IgG. The antibody assay was performed according to the manufacturer’s instructions. Briefly, magnetic beads conjugated with antibody isotypes were seeded into a 96-well plate. Antibody standards were serially diluted in a 1:3 serial dilution in universal buffer, constructing a 7-point standard curve, in addition to a blank made of universal buffer. BALF and serum were then added in duplicates (1:2 dilution and 1:20,000 dilution in universal buffer, respectively) into appropriate wells containing the magnetic beads. The plate was placed in a hand-held magnetic plate washer, and shaken with the thermomixer comfort (Eppendorf, Hamburg, Germany at 500 RPM for 60 mins. The plate seal was removed, wells were washed thoroughly using wash buffer and detection buffer was added to each of the wells. The plate was sealed and placed on the shaker table for a further 30 mins at 500RPM at room temperature.The plate was then analysed by a Magpix® multiplex reader (Luminex, Austin, USA).

### Statistical analysis

Data were represented as the mean ± standard error of the mean (SEM). Body weight was analysed using two-way analysis of variance (ANOVA) followed by Tukey’s *post hoc* test for multiple comparisons. Airway inflammation, ROS production, cytokine mRNA expression and antibody titres were analysed using one-way ANOVA followed by Tukey’s *post hoc* test for multiple comparisons. Viral titres were analysed using an unpaired student’s t test. All tests were performed by Graphpad Prism 7.0b (San Diego, CA, USA) and statistical significance was taken at p < 0.05. N is representative of different samples from each mouse.

### Chemicals

Aldara 5% (Meda®, Solnam, Sweden) was stored at room temperature, and weighed to 25 mg when required. Imiquimod (Invivogen, Thermo Fisher Scientific, Carlsbad, USA, cat. no. tlrl-imq) was dissolved in PBS at a concentration of 1 mM and aliquoted into tubes containing 0.5 mL. L-012 (WAKO chemical, Richmond, USA, cat. no. 120-04891) and phorbol 12,13-dibutyrate (PDB; Sigma Aldrich, St Louis, USA, cat. no. P1269-5MG) were dissolved in dimethyl sulfoxide (DMSO; Sigma Aldrich, St Louis, USA, cat. no. 472301; 100%) in 10 μL and 5 μL aliquots, respectively at a concentration of 10^−2^M. Chemicals were stored at −20 °C and quickly thawed when required.
